# Nanotechnology in Wastewater Management: A New Paradigm Towards Wastewater Treatment

**DOI:** 10.3390/molecules26061797

**Published:** 2021-03-23

**Authors:** Keerti Jain, Anand S. Patel, Vishwas P. Pardhi, Swaran Jeet Singh Flora

**Affiliations:** 1 Department of Pharmaceutics, National Institute of Pharmaceutical Education and Research (NIPER)—Raebareli, Lucknow 226002, India; aspsidhi1998@gmail.com (A.S.P.); vishwaspardhi678@gmail.com (V.P.P.); 2 Department of Pharmacology and Toxicology, National Institute of Pharmaceutical Education and Research (NIPER)—Raebareli, Lucknow 226002, India

**Keywords:** wastewater treatment, nanotechnology, adsorption and biosorption, nanofilters, photocatalysis, disinfection, sensors, carbon, metals, zeolites

## Abstract

Clean and safe water is a fundamental human need for multi-faceted development of society and a thriving economy. Brisk rises in populations, expanding industrialization, urbanization and extensive agriculture practices have resulted in the generation of wastewater which have not only made the water dirty or polluted, but also deadly. Millions of people die every year due to diseases communicated through consumption of water contaminated by deleterious pathogens. Although various methods for wastewater treatment have been explored in the last few decades but their use is restrained by many limitations including use of chemicals, formation of disinfection by-products (DBPs), time consumption and expensiveness. Nanotechnology, manipulation of matter at a molecular or an atomic level to craft new structures, devices and systems having superior electronic, optical, magnetic, conductive and mechanical properties, is emerging as a promising technology, which has demonstrated remarkable feats in various fields including wastewater treatment. Nanomaterials encompass a high surface to volume ratio, a high sensitivity and reactivity, a high adsorption capacity, and ease of functionalization which makes them suitable for application in wastewater treatment. In this article we have reviewed the techniques being developed for wastewater treatment using nanotechnology based on adsorption and biosorption, nanofiltration, photocatalysis, disinfection and sensing technology. Furthermore, this review also highlights the fate of the nanomaterials in wastewater treatment as well as risks associated with their use.

## 1. Introduction

For survival and development of living beings, many things are required, but no other things could be more important than water. Earth is known as a blue planet as about 70% of the earth’s surface is covered with water. Saline water makes about 97.5% of the total water, while the remaining 2.5% is regarded as fresh water and out of this fresh water about 68.9% of water is in the form of ice, permanent snow, and glaciers. Furthermore, ground water accounts for 30.8% of fresh water, out of which only 0.3% is easily accessible [1]. Clean and safe water is a basic asset for a flourishing society as well as a thriving economy [2]. Unfortunately, a rapid increase in population, expanding industrialization, urbanization and extensive agriculture practices are causing continuous deterioration of quality water resources, which is a serious matter of global concern [3,4,5]. Worldwide, around 1.2 billion populations do not have access to safe drinking water, 2.6 billion people struggle to fulfil basic sanitation, millions of people, particularly children, have lost their lives from diseases communicated through unsafe and polluted water [6,7]. Diarrhea caused by the consumption of contaminated water takes the life of approximately 1.8 million children every year [6,8].

Physicochemical or conventional and biological methods to address wastewater treatment include coagulation, flocculation, precipitation, adsorption, ion-exchange, electro-dialysis membrane separation, and aerobic, anoxic or anaerobic oxidation methods. However, wastewater treatment through these physicochemical methods often involve chemicals (such as chlorine compounds, ammonia, permanganate, alum, sodium hydroxide, hydrochloric acid, ozone, and ferric salts, coagulation and filtration aids, ion exchange resins and regenerants) and energetically and operationally intensive mechanical methods, and thus requires engineering expertise and infrastructure. Additionally, it has been observed that conventional techniques are not efficient enough to remove toxins, phosphorous, nitrogen, heavy metals completely from contaminated wastewater. Although all these factors rendered them expensive and time consuming, all of them decrease the level of various pollutants to some extent and have their own advantages and disadvantages [6,7,9,10].

Nanotechnology is basically a manipulation of matter at the molecular and atomic levels to craft a new structure, device and system with superior electronic, optical, magnetic, conductive and mechanical properties [11,12,13,14,15]. Nanotechnology is being explored as a promising technology, and has demonstrated remarkable accomplishments in various fields including wastewater treatment. Nanostructures offer unparalleled opportunities to make more effective catalysts and redox active media for wastewater purification, owing to their small size, large surface area, and ease of functionalization. Nanomaterials have been found to be effective in elimination of several pollutants from wastewater such as heavy metals, organic and inorganic solvents, color as well as biological toxins, and pathogens that cause diseases like cholera and typhoid [6].

## 2. Wastewater: Sources and Composition

**Figure 1:**
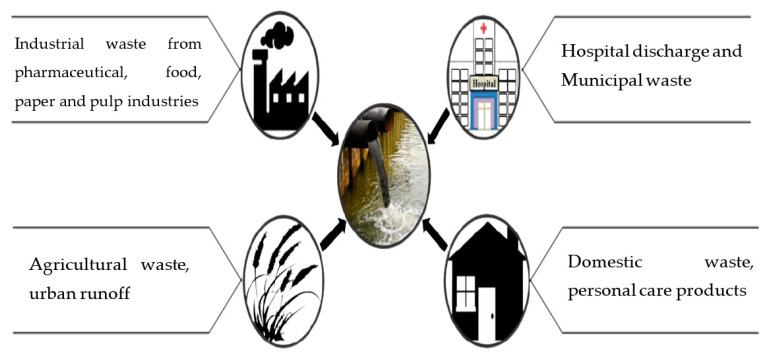
Various sources of wastewater.

Wastewater contains several dangerous and harmful materials and it originates from various sources, which includes sewage, industrial and commercial waste, agricultural waste etc., which could be characterized by their physical appearance, chemical composition, and loads of microorganisms [16]. Generally, wastewater comes from normal living processes or in other terms wastewater is any water that has been contaminated by human use [17,18]. The major sources of wastewater are domestic wastewater, agricultural waste, industrial waste, and commercial waste [19]. Almost all the major sources need good quality water, particularly industries, but in return a huge volume of contaminated and polluted water is generated and streamed in large water bodies, making them polluted [20,21]. Different sources of wastewater are shown in Figure 1.

**Figure 2:**
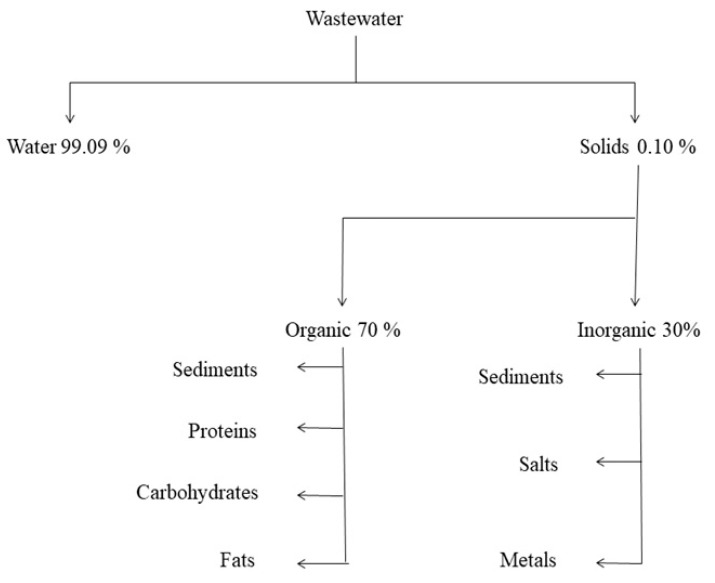
Typical composition of sewage water.

Wastewater is a complex matrix composed of 99.9% of water and the remaining 0.1% includes suspended solids (350–1200 mg/L), organic compounds like body waste (i.e., feces, toilet paper, food waste), dissolved biodegradable organics (i.e., proteins, carbohydrates and lipids), inorganic solids (i.e., sediment soil, salts and metals), and particulate stuff with an chemical oxygen demand of 250–1000 mg/L, several microorganisms (up to 10^9^ number/mL), heavy metals, micro-pollutants and nutrients. Almost, 63% of phosphate compounds have been found to be associated as a soluble fraction in wastewater [22,23]. Figure 2 shows the typical wastewater composition.

## 3. Common Steps in Wastewater Treatment

In recent years, there has been a rise in industrialization and population growth. In the meantime, the rate of wastewater generation has also grown, which became a serious matter of concern for the environment and ecosystems as well. Accordingly, a proper wastewater treatment is required in order to reuse or return the water to the environment [24]. A process of separation of pollutants or contaminants from wastewater by taking aid of physical or chemical processes before releasing them in the environment is known as wastewater treatment. The aim of wastewater treatment is not only to eradicate contaminants and pollutants below the maximum allowed limit, but also to recover micronutrients and water to avoid environmental and human health threats [25,26]. The biological wastewater treatment of domestic wastewater can be divided into two main aerobic processes; that is, suspended growth and fixed film processes. Activated sludge is the most widely used suspended growth system, either in the most conventional configuration or as an oxidation ditch or sequential batch reactor, among others. The anaerobic treatment involves anaerobic bacteria that transforms the organic matter present in the wastewater into biogas, which contains large amounts of methane gas and carbon dioxide. The anaerobic treatment is preferred when the dissolved organic concentrations of untreated wastewater are high [21]. The number of stages required to treat wastewater greatly depends on the extent of pollutant to be eliminated and the mechanism of elimination [27].

Preliminary treatment is the first step towards wastewater treatment, which removes large and/or heavy debris. Preliminary treatment typically takes place in two stages: screening followed by grit removal. The screening process removes large floating debris such as rags that account for ~60% of the debris, paper ~25% and plastics ~5%, by using screens. Grit removal is the immediate step followed by screening which chiefly removes inorganic particles like gravel, sand and other heavy particulate matters (e.g., bone fragments, coffee grounds and corn kernels) by settling in grit channels [23]. After preliminary treatment, the effluent goes to primary treatment, which predominantly separates the suspended solids via a sedimentation process. Sedimentation takes place in tanks where the effluent is allowed to stay for several hours so that the suspended solids either get settled down or form smut on the top, which is skimmed from the top, and sludge (settled solid on the bottom of the tank) is removed. Primary treatment removes around 40% of biological oxygen demand (BOD), about 80–90% of suspended solids, and around 55% of fecal coliforms [28].

The secondary treatment involves the processing of activated sludge from primary treated water via oxidation ditches, trickling filters or bio-filters, and rotating biological contactors. A combination of the above-mentioned processes in a series is used in the treatment of municipal wastewater having a significant amount of organic materials. To oxidize these organic contaminants, the aid of millions of actively growing microorganisms (single cell), particularly bacteria and protozoa, is taken and they remove organic contaminants from wastewater as a result of their natural metabolic activity [29]. Along with the removal of organic contaminants, secondary treatment also removes some micronutrients such as nitrogen and phosphorous from sewage by the process of nitrification and luxury cell uptake. These micronutrients are usually taken up by algae and fungi for their growth through the process of eutrophication, resulting in diminution of the oxygen level of the water body to which the treated water is discharged [30]. In one study, Yamashita and Ryoko had investigated the efficiency of anoxic bioreactors packed with various combinations of wood and iron along with trickling filters packed with ceramics for the process of nitrification to remove nitrogen and phosphorous from wastewater over a long operation. They found nitrogen and phosphorous removal performance of the bioreactor packed with aspen wood and iron, higher than that of bioreactor packed with cedar chips and iron, during the operational period which was found to continue over a period of 1200 days [31].

Tertiary treatment involves the removal of residual organic, inorganic matter and microorganisms from the effluent of secondary treatment and disinfection of treated sewage by treatment with chlorine, chlorine dioxide, sodium hypochlorite and chloramines, UV (ultra-violet) or ozone radiation before releasing in the environment to make sure that the treated sewage is safe enough to be released [28,30]. It is important to note that the abovementioned stages or processes of wastewater treatment are the basic and traditional ones and the advanced methods based on nanotechnology have been discussed in detail in the following sections.

## 4. Nanotechnology in Wastewater Management

There are various types of nanomaterials reported, which could be used in the wastewater treatment such as polymeric nanoparticles (NPs), metal NPs, carbon-based nanomaterials, zeolite, self-assembled monolayer on mesoporous supports (SAMMS), biopolymers and many more [32]. Nanotechnology-based pathways, which are being employed for wastewater remediation, are adsorption and biosorption, nanofiltration, photocatalysis, disinfection and pathological control, sensing and monitoring and so on.

### 4.1. Adsorption and Biosorption

Adsorption is an exothermic process and a surface phenomenon which involves the process of the transfer of a phase (a molecule or ion present in either liquid or gaseous bulk) called adsorbate, onto a solid, rarely liquid surface called an adsorbent to form a monomolecular layer on the surface via physicochemical or chemical interactions under specific conditions [33,34,35]. Biosorption is a kind of adsorption in which biological materials such as certain type of bacteria, algae or fungi act as adsorbents due to their intrinsic property to bind and mount up heavy metals, even from a very dilute aqueous solution or via metabolically mediated (by making use of ATP) or spontaneous physicochemical pathways of uptake (not at the cost of ATP). The process of biosorption principally involves microprecipitation, ion exchange and cell-surface complexation [36,37,38,39].

Yang et al. investigated the biosorption of chromium (Cr) (VI) from synthetic wastewater using algal-bacterial aerobic granular sludge. They found that biosorption of Cr(VI) was highly depended on pH, and observed that maximum Cr(VI) biosorption capacity of algal-bacterial aerobic granular sludge was 51.0 mg g^−1^ at pH 2. Cr(VI). Removal was predominated by biosorption accompanied with bioreduction. Desorption with NaHCO_3_ could recover 64–73% of the adsorbed Cr and most of this was in the form of Cr(III). Compared to the conventional bacterial aerobic granular sludge, algal-bacterial aerobic granular sludge demonstrated a higher biosorption capacity and a better granular stability [40]. Similarly, Ding et al. prepared alginate-immobilized *Aspergillus niger* microsphere (AAM) biosorbent for removal of thorium (Th) ions, particularly Th(IV), from radioactive wastewater. AAM exhibited a superior (303.95 mg·g^−1^) biosorption performance at pH 6 and 40 °C. Rapid Th(IV) enrichment can be achieved in less than 100 min [41]. 

#### 4.1.1. Carbon-based Nano-Adsorbents

In various fields of science and technology, carbon science has been studied for decades. Nanostructures of carbon are known to have different low-dimension allotropes of carbon such as activated carbon, carbon nanotubes (CNTs), and the C_60_ family of buckyballs, graphite, and graphene [42]. Carbon nanostructures are widely used as nanoadsorbents for wastewater treatment owing to their abundant availability, cost-effectiveness, high chemical and thermal stabilities, high active surface areas, excellent adsorption capacities, and environmental friendly nature [43]. For years, activated carbon is being used as the most common adsorbent due to their high porosity and large surface area. Although, high cost confines their use, therefore different allotropes of carbon and functionalized carbon are being examined as nanoadsorbents [44].

CNTs are cylindrical, bulky molecules comprised of hybridized carbon atoms in hexagonal assortment, which may be produced by rolling up single or multiple sheets of graphene in order to make single-walled CNTs (SWCNTs), and multiple-walled CNTs (MWCNTs), respectively [45]. Researchers are showing great interest towards CNTs because of their exceptional properties, such as their mechanical flexibility, high specific surface areas, and large pore volumes and that is why CNTs are extensively being exploited in wastewater treatment [46,47,48]. As adsorbents, these CNTs have shown superior performance over other adsorbents attributed to tunable surface chemistry, which permits surface modifications, a chemically inert nature, hollow structure, high specific surface area, light mass density, high porosity and strong interaction with pollutants [49]. All these properties make them excellent to be utilized in wastewater treatment [50]. Heavy metals and ions present in water are a serious peril to the environment as well as human health. Yadav and Srivastava investigated adsorption and desorption of Mn^7+^ ions by CNTs and observed that CNTs adsorb Mn^7+^ efficiently and UV-Visible spectrophotometric analysis revealed that CNTs brought down its concentration from 150 ppm to 3 ppm. They have used laboratory grade KMnO_4_ as a source of Mn^7+^ ions [51].

Demand of pharmaceutical products has grown a lot as a result of population growth and widespread illness. Antibiotics are one of the most widely consumed pharmaceutical product and thus their discharge in the surrounding ecosystem has also increased. Such products, when consumed by humans or other living organisms, cause serious health issues. Kariim et al. synthesized MWCNTs adsorbent by making use of activated carbon derived from wood sawdust, and doped it with nickel-ferrites (Ni-Fe) for the sorption of metronidazole and levofloxacin from pharmaceutical wastewater. The surface morphological analysis revealed the surface area of pure activated carbon and Ni-Fe supported activated carbon CNTs, which was 840.38 and 650.45 m^2^/g, respectively. Results from the adsorption process demonstrated a high adsorption capacity of developed MWCNTs for metronidazole and levofloxacin [52]. Zhao and coworkers prepared magnetic MWCNTs (MMWCNTs) that possess the property of both magnetic NPs and CNTs for the removal of tetracycline (TC). The prepared MMWCNTs exhibited the size of 10–50 nm, magnetic separability (10.8 emu/g), and high adsorption capacity (*qm* =4 94.91 mg/g, 308 K). MMWCNTs showed >80% efficiency in adsorptive removal of TC in the pH range of 2–10, when the TC concentration was less than 80 mg L^−1^, as determined in batch experiments including adsorption kinetics, adsorption isotherms, and the effect of the initial pH on TC adsorption by the MMWCNTs. The well-fitted Langmuir isotherm and pseudo-second-order dynamic adsorption model indicated that the adsorption of TC onto MMWCNTs principally involved chemical and monolayer adsorption [53]. Similarly, Yang and coworkers developed surface oxidized nano-cobalt magnetic nanomaterial embedded with nitrogen-doped CNTs (Co@CoO/NC) for effective adsorption of TC and rhodamine B (RhB). The optimized Co@CoO/NC showed an excellent adsorption capacity for both RhB (679.56 mg⋅g^−1^) and TC (385.60 mg⋅g^−1^). Being a recyclable and reusable magnetic adsorbent, Co@CoO/NC maintained 75% and 84% of adsorption capacities for TC and RhB, respectively, after four repetitions [54].

In 2017, Bankole et al. put an effort to make purified CNTs functionalized with polymers as a nanoadsorbent to remove chemical oxygen demand (COD) from wastewater coming from the electroplating industry. Various polymers, such as amino polyethylene glycol (a@PEG), polyhydroxylbutyrate (PHB), purified CNTs (P-CNT), and amino polyethylene glycol with polyhydroxylbutyrate (a@PEG-PHB) were used to functionalize the prepared MWCNTs. Each of the functionalized MWCNTs was allowed to equilibrate for the time of 70 min and then evaluated for an order of maximum COD removal and the order was a@PEG-CNTs (99.68%) > PHB-CNTs (97.89%) > P-CNTs (96.34%) > a@PEG-PHB-CNTs (95.42%). Sorption equilibrium data were best described by Freudlich isotherm with the correlation coefficient of R^2^ > 0.92 than Langmuir isotherm. The adsorption kinetics for COD removal from wastewater coming from electroplating industry fitted best to the pseudo-second-order model with rate constant within the range of 4 × 10^−5^ to 1 × 10^−4^ (g·mg^−1^ min^−1^) [55].

**Figure 3:**
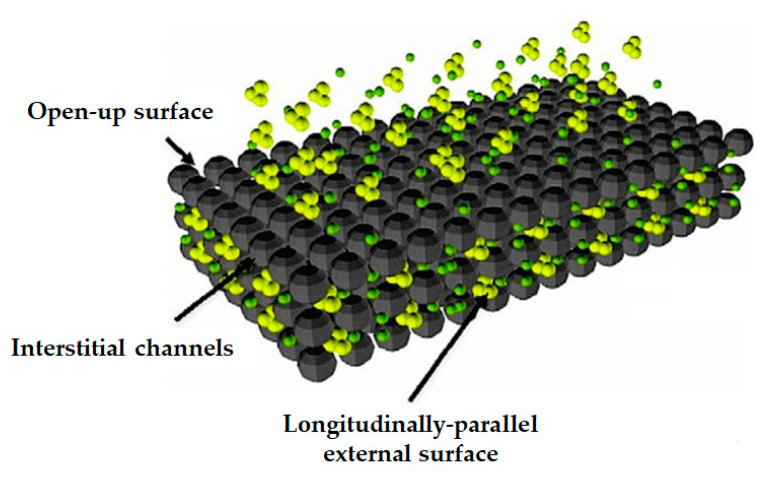
Graphical representation of structure and absorption sites of graphene sheets (Reproduced with permission from [65]).

Graphene is a two-dimensional material composed of sp^2^ hybridized carbon atoms compiled in a hexagonal assortment and exhibits ambipolar electric field effects, classical thermal conductivity and quantum hall effects at room temperature, and possesses high surface area and porosity, which makes it an excellent candidate to adsorb several gases like methane, hydrogen, and carbon dioxide. A high surface area, increased active sites, huge delocalized π-electron systems, and good chemical stability render it suitable to be utilized as an adsorbent for wastewater handling (Figure 3). Properties of this material can be modulated by altering the layer’s number and stacking [56,57]. Graphene oxide (GO) is a single monomolecular layer of graphite with a variety of oxygen holding functionalities such as hydroxyl, carboxyl, carbonyl, and epoxide groups [58]. Currently, GO, along with magnetic particles, has gained considerable attention as an adsorbent for wastewater treatment because of its simple design, lower sensitivity, low cost and ease of operation towards the toxic pollutants [59,60]. Chen et al. envisaged the preparation of aerogel (AG) of GO/aminated lignin (GO/AL-AG) for adsorption of malachite green (MG) dye in wastewater and compared the adsorption capacity at different aerogels dosage, pH, contact time and reaction temperatures. The maximum adsorption capacity and efficiency of prepared GO/AL-AG was revealed by experimental results and was found to be 113.5 mg/g and 91.72% under the optimal conditions. The adsorption performance of GO/AL-AG was enhanced significantly in comparison with other AG in the experiment and this was attributed to the synergy between the carboxyl group present over the surface of GO and the amine of aminated lignin. The adsorption efficiency of the GO/AL-AG was about 90% within 5 cycles of adsorption–desorption [61]. Similarly, Bu et al. developed GO functionalized by thiosemicarbazide (TSC) (GO-TSC) as an adsorbent material for efficient removal of methylene blue (MB) from wastewater. The GO, and GO-TSC had high stability with maximum adsorption capacity of 196.8, and 596.642 mg/g, respectively [62]. Zheng and co-workers prepared hydrogel of GO decorated with silver nanoparticles (Ag NPs) followed by integration with a porphyrin complex as a wastewater remediation technique for adsorption of dyes present in wastewater. Hydrogels were modified with different porphyrin complexes and were evaluated for their adsorption capacity and it was found that tetraphenylporphyrin-modified hydrogel exhibited the highest adsorption capacity (130.37 mg/g) for MB [63]. Recently, Sirajudheen et al. fabricated a hydrocomposite (HCP) of GO supported by a biopolymer, namely chitosan (CS) (GO/CS-HCP) for the efficient removal of organic pollutants from wastewater. The fabricated GO/CS-HCP showed the maximum adsorption capacity for Congo Red (CR) (43.06 mg/g) followed by Acid Red 1 (AR1) (41.32 mg/g) and reactive Red 2 (RR2) (40.03 mg/g) dyes in wastewater. Enhanced adsorption was observed at pH 2 and the removal efficiency decreased with an increase in the pH. Prepared GO/CS-HCP showed an ideal desorption, with more than a 65% regeneration ability in the 0.1 M NaOH solution [64].

#### 4.1.2. Metal based Nanoadsorbents

Currently, metal-based nanomaterials as adsorbents are catching the eyes of researchers [66]. Nanometals and their oxides such as Fe_3_O_4_ [67]_,_ TiO_2_ [68], MnO_2_ [69], MgO [70], ZnO [71] and CdO [72] are extensively used to remove heavy metals, ions and dyes from wastewater [73]. Nanometal oxides are considered more effective adsorbent as compared to activated carbon when removal of heavy metals and radioactive metals are concerned. Additionally, their small size and large surface area offers a small intraparticle diffusion distance which can be compressed without altering their surface area. The sorption process is mainly governed by the complexation between dissolved metals and the oxygen in metal oxides [74,75].

Wastewater coming from oil refinery contains a variety of ions and metals such as calcium (Ca^2+^) and copper (Cu^2+^). To remove such metals and ions, He et al. prepared reusable nanoadsorbents based on Fe_3_O_4_/GO-COOH by the magnetization and carboxylation of GO. The nanoadsorbents showed 78.4% and 51% percent removal of Ca^2+^ and Cu^2+^ respectively, at 60 min. The nanoadsorbent retained high recovery rates (82.1% for Ca^2+^ and 91.8% for Cu^2+^) and removal percentages (72.3% for Ca^2+^ and 49.33% for Cu^2+^) after five adsorption–desorption cycles [76].

Peralta et al. developed magnetic nanoadsorbents based on silica and evaluated its potential for the elimination of organic pollutants such as contaminants of emerging concern (CECs), polyaromatic hydrocarbons (PAHCs), and aliphatic hydrocarbons (AHCs). The preparation process included the covering of magnetic iron oxide NPs with a hybrid shell consisting of silica and 3-(trimethoxysilyl)propyl-octadecyldimethyl-ammonium chloride (3-TPODAC) as a structure-directing agent. The prepared magnetic mesostructured silica NPs (MMSSNPs) were further modified by means of trimethoxyphenylsilane, to get nanoadsorbents functionalized with phenyl (MMSSNPs-ph). Both the materials were characterized and evaluated for batch sorption tests with single and a mixture of contaminants, and the results revealed that MMSSNPs-ph is more proficient for the adsorption of PAHCs and AHCs. The presence of phenyl and 3-TPODAC moieties on the mesostructured silica scaffolds was found to be a key factor in obtaining high PAHCs uptake from aqueous media. The prepared MMSSNPs-ph was also tested against CECs such as carbamazepine, diclofenac, and ibuprofen and the result showed that although MMSSNPs had better adsorption capacities of CECs, MMSSNPs-ph attained high ibuprofen and diclofenac uptakes [77].

Sadak., et al. fabricated polyacrylic acid (PAA) conjugated ferric oxide (Fe_3_O_4_) magnetic NPs (MNPs), which were further functionalized with CR azo dye (PAA-CR/MNPs). This PAA-CR/MNPs system showed a binding affinity for various cations such as Fe^2+^, Fe^3+^, Cd^2+^, Cu^2+^, and Pb^2+^. Heavy metal removal efficiency of PAA-CR/MNPs was investigated at various pHs, temperatures, reaction conditions, and times with a special emphasis on Pb^2+^. The removal efficiency and adsorption capacity of PAA-CR/MNPs for Pb^2+^ were found to be maximal at 6.5 pH and 45 min of reaction time, and the Pb^2+^adsorption kinetics were best fitted to a pseudo second-order model [78].

Arshadi et al. synthesized an adsorbent based on sineguelas waste (S-NaOH) from agriculture biomass, further decorated with nanoscale zerovalent iron particles (NZVIPs). The fabricated system was subjected for the investigation of adsorption/reduction of inorganic pollutant such as Pb^2+^ ions. The NZVIPs showed good dispersion (ca.10–70 nm) over the surface of sineguelas waste. The fabricated system had a feasible and spontaneous adsorption profile for Pb^2+^ [79]. Jethave and coworkers developed an efficient nanoadsorbent that consisted of zinc-aluminium oxide NPs doped with lead (LD/Zn-AlO/NPs) for adsorption of anionic dyes such as methyl orange (MO). The MO removal efficiency of LD/Zn-AlO/NPs reached 99.60% after 30 min. The spontaneous and exothermic nature of adsorption was indicated by thermodynamic parameters. LD/Zn-AlO/NPs showed a maximum adsorption capacity of 200 mg/g for MO in a single component system [80].

#### 4.1.3. Polymer-Based Nanoadsorbents

Over the last several years, extensive research has been done on polymer nano composites for the improvement of environmental sustainability and for wastewater treatment. It offers high surface area for fast decontamination, improved processability, remarkable stability, improved processability, cost effectiveness, selectivity to eliminate different pollutants in wastewater [81,82]. Cost-effective and commonly used polymeric adsorbents include polysaccharides, namely CS cyclodextrin, nano-magnetic polymers, covalent organic polymers, extracellular polymeric substances etc. [83,84] Nanocellulosics, which are derived from cellulose, possess the advantages of being nontoxic, ubiquitous, excellent adsorbents, and have an ease of surface modification, rendering them suitable for wastewater remediation [85]. Recently, preparation of lignin-derived nanomaterials have been introduced, and have demonstrated remarkable potential for water/wastewater treatment. They have been found effective in the catalytic degradation of dyes, nitroarenes, and the removal of heavy metals [86].

Abdi and Abedini developed a metal organic framework adsorbent of zeolitic imidazolate framework (ZIF) based on polymeric nanocomposite beads, namely polyether sulfone (PES), for the efficient removal of Malachite green (MG) from wastewater. The influence of various parameters such as pH, adsorbent dosage, and initial MG concentration were examined on the developed PES-ZIF-8/ZIF-67, as well as PES beads alone. The result showed that PES beads alone had negligible adsorption, whereas PES-ZIF-8/ZIF-67 had an adsorption capacity of 613.2 mg/g, with an MG removal efficiency of 99.2%. The adsorption mechanism was attributed to the aromatic–aromatic interaction (π-π stacking) among the rings of MG dye and imidazole ligand [87].

Chen and coworkers fabricated bi-functionalized β-cyclodextrin (β-CD) and polyethyleneimine (PEI) magnetic nanoadsorbents (β-CD/PEI- Fe_3_O_4_) in order to capture MO and Pb^2+^ simultaneously from wastewater with spatially separated sorption sites. In the constructed system, N-bearing groups of PEI, with a positive charge and cavities of β-CD, were thought to be principally responsible for MO removal via electrostatic attraction and host–guest inclusion, respectively. The edges of β-CD with oxygen bearing groups and free amino moieties present on the PEI, acted as active sites for the efficient uptake of Pb^2+^ from wastewater [88]. Moharrami and Motamedi constructed a nanocomposite hydrogel of biological origin by making use of starch-grafted copolymers of 2-acrylamido-2 methyl propane sulfonate and acrylic acid {S-g-(AMPS-co-AA)} with the further addition of cellulose nanocrystals functionalized with magnetite (M-CNCs) for adsorption of cationic dyes such as crystal violet (CV) and MB. The fabricated system showed an adsorption capacity of 2500.0 mg/g and 1428.6 mg/g for CV and MB, respectively [89].

Priya et al. envisaged the preparation of nanoadsorbents based on iron–aluminum (Fe-Al) layered dual hydroxide/reduced GO (rGO) coated with sodium alginate (SA) (FAH-rGO/SA) for removal of arsenic (As). The prepared FAH-rGO/SA-4, FAH- rGO -/SA-1 and FAH-rGO were evaluated for maximum adsorption capacity, which was found to be 190.84, 151.29 and 115.39 mg/g, respectively. The overall results demonstrated a high removal efficiency (>98%) of FAH-rGO for As (V) and this was attributed to a high weight percentage of SA [90]. Oilfield-produced water has a high salinity due to the presence of metal ions such as Ca^2+^ and Mg^2+^. To address this issue, He and coworkers made nanoadsorbents composed of poly (ethylene glycol) (PEG), aminated GO (NH_2_-GO), magnetic Fe_3_O_4_ NPs (PEG/Fe_3_O_4_/NH_2_-GO). The prepared PEG/Fe_3_O_4_/NH_2_-GO system showed 69.8% and 61.1% removal ratios at 10 min for Ca^2+^ and Mg^2+^, respectively. Overall results indicated its reusability and stability for oilfield wastewater treatment [91].

#### 4.1.4. Zeolites

Zeolites are three-dimensional, crystalline microporous materials, having well-defined structures of voids and conduits of distinct size, which is easily accessible through pores of well-defined molecular dimensions that hold aluminum, silicon and oxygen in their normal framework [92,93,94]. Zeolites are found naturally as silicate minerals and can also be prepared synthetically as magnetically modified zeolite, and bio-zeolite, etc. [95] Presently, zeolitic nanomaterials as adsorbents have attracted considerable attention in environmental applications due to their stability in water, their low cost of production, high surface area, selectivity and compatibility with the natural environment [96,97,98]. So far, numerous studies have confirmed their extraordinary performance for the removal of metal cations from wastewater [99]. Zeolite is found to possess numerous research applications in industries, its adsorbent property is most explored due to their ability to regenerate and reuse, which renders them suitable candidates for wastewater treatment [100,101]. Zhao et al. prepared cubic NaA zeolite, which is one of microporous crystalline aluminosilicate zeolite composed of Na_2_O/Al_2_O_3_. It is obtained from natural halloysite minerals by means of nanotubular structures as a source material for adsorption of ammonium ions (NH_4_^+^) from wastewaters. The maximum adsorption capacity of prepared NaA zeolite for NH_4_^+^ ions was found to be 44.3 mg/g. The constructed adsorbent system showed reusability and demonstrated a potential application in the elimination of NH_4_^+^ pollutants from wastewater [98].

Bandpi and coworkers fabricated an adsorbent based on natural zeolite (NZ) coated with Fe_3_O_4_ (CNZ) nanoparticles for cephalexin (CEX) removal from aqueous solution. The maximum CEX removal efficiency for CNZ, and NZ were found to be 93% and 28%, respectively [102]. Similarly, Samarghandi et al. fabricated manganese oxide NPs coated with NZ adsorbents for CEX removal form aqueous solution. The evaluated maximum CEX removal efficiency was 89% and 28% for CNZ and NZ, respectively at the pH 7 [103]. Esmaili and Saremnia developed NaA zeolite NPs (NaA-z@NPs) from the husk of *Hordeum vulgare L.* for adsorption of total petroleum hydrocarbon (TPH). TPH removal efficiency of NaA-z@NPs at optimal conditions in both batch and continuous systems was observed to be 92.3% and 87.4%, respectively [94].

Gugushe et al. synthesized a nanocomposite of MMWCNTs further coated with zeolite (Fe_3_O_4_-MWCNTs/Zeolite) for adsorption of Pb and thallium (TI) in complex environmental samples. The synthesized Fe_3_O_4_-MMWCNTs/Zeolite was utilized as an adsorbent in ultrasonic-assisted magnetic solid phase extraction for Pb and TI, which showed maximum adsorption capacity of 37.8 and 44.5 mg·g^−1^, respectively [104]. Nyankson et al. fabricated zeolite and zeolite nanocomposite (Zeolite-Fe_3_O_4_@NC) for adsorption of organic molecules such as MB from solution. The synthesized Zeolite-Fe_3_O_4_@NC was examined for its potential to adsorb MB from solution by taking aid of UV-visible and kinetic and equilibrium isotherm models. The maximum adsorption capacity and efficiency of Zeo-Fe_3_O_4_@NC was 2.57 mg/g and 97.5%, respectively, at 25 °C and after regeneration, the maximum adsorption efficiency at a pH of 7 was found to be 82.6% [105].

### 4.2. Nanofilters

Water filtration is the process of stripping or lowering down the concentration of particulate matter, such as suspended particles, microorganisms as well as other detrimental biological and chemical contaminants from contaminated or polluted water to make safe and clean water for drinking, pharmaceutical and medical applications [106]. Membrane technology has gained considerable attention in recent years and the most important advancement in membrane technology is nanofiltration (NF) membrane. As the name itself suggests, NF membranes have a molecular weight cut-off (MWCO) for uncharged particles in the nanometer range [107]. NF membranes are relatively recent and are the most preferably used method for drinking water and wastewater treatment [108]. NF is a pressure-driven membrane process that lies between ultrafiltration (UF) and reverse osmosis (RO), with a pressure between 5–20 bars and a pore size between 0.5 and 2.0 nm characterized by a high rejection of divalent or higher-valent ions, a low rejection of monovalent ions, and high flux and low energy consumption compared to RO, and a high rejection compared to UF [109,110,111,112,113]. It is quite a recent advancement in membrane technology and it can be aqueous or non-aqueous. NF is one of the most significant and widely employed techniques in the field of wastewater treatment attributed to its exclusive filtration mechanism and the availability of a variety of membranes. NF is appropriate to filter out more or less all organic and inorganic contaminants, including quite a lot of harmful microbes from wastewater [114,115,116]. NF membranes are highly flexible, cost-effective and easy to produce. Two types of NF membranes are most commonly used, including polymeric membranes and ceramic membranes. The polymeric membranes display a short lifetime due to their inferior chemical resistance and a high fouling rate [117]. On the other hand, ceramic membranes have higher mechanical, chemical and thermal stabilities [118]. 

Mostafavi et al. fabricated an NF based on CNTs for the removal of MS2 virus from water. The MS2 virus is a member of the family related to bacterial viruses which infects the bacterium *Escherichia coli* and other members of the *Enterobacteriaceae* family. The NF was characterized for its porosity and surface morphology and then evaluated for MS2 virus removal efficiency. The result demonstrated high removal efficiency at 8–11 pressure bars [119]. Similarly, Parham et al. fabricated a filter based on CNT-ceramic composite for yeast filtration. The fabricated filter demonstrated high (98%) filtration efficiency for yeast, and almost a 100% removal efficiency for heavy metal ions from water [120]. Han and coworkers prepared an NF membrane on the mesoporous substrate for water purification. The fabricated filtration membrane had a thickness of 22–53 nm and demonstrated an efficient retention for organic dye present in the water. However, the moderate retention was found for ionic substances [121]. Similarly, Nair et al. made a submicrometer-thick NF membranes that displayed an exceptionally high impermeability to any kind of vapors, gases and liquids, excluding water [122].

### 4.3. Photocatalysis

**Figure 4:**
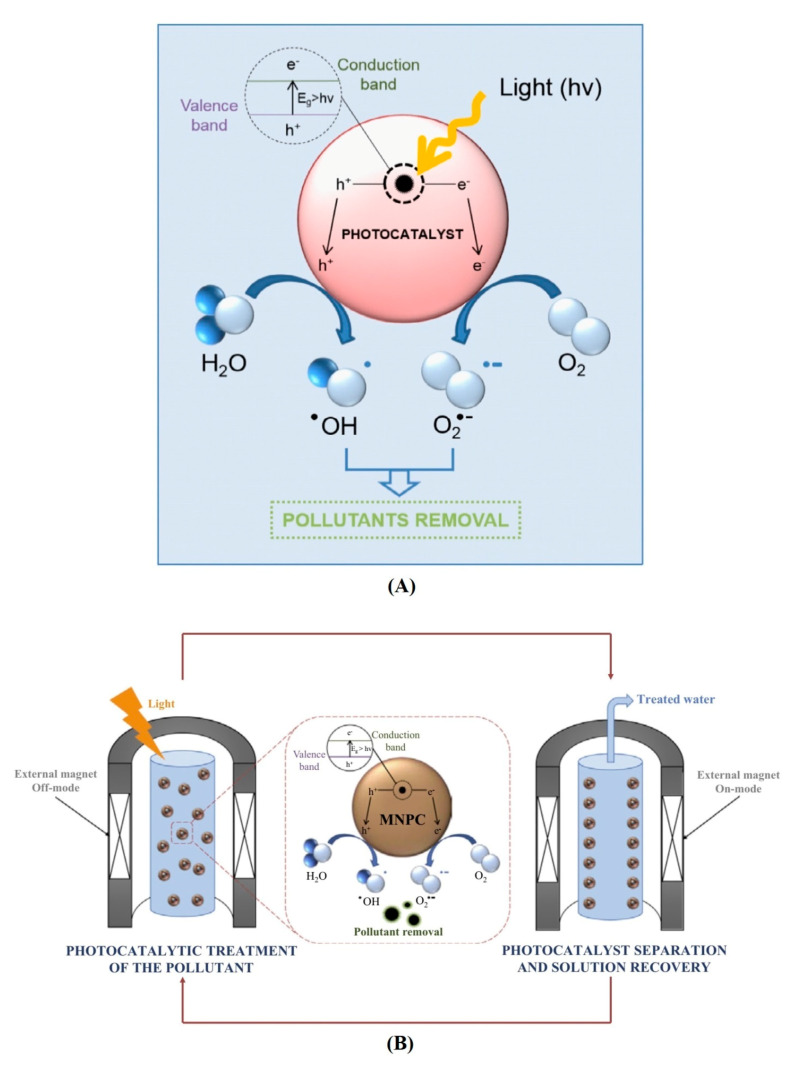
Graphical representation of (**A**) excitation of a nanophotocatalyst during the photocatalytic process; (**B**) photocatalytic treatment of polluted water and recovery of nanophotocatalyst (Reproduced with permission from [130]).

The term “Photocatalysis” comprises two Greek words. “Photo” means “light” and “catalysis” means any substance that alters the rate of a chemical reaction without being involved in the reaction. Therefore, photocatalysis can be defined as a light-induced reaction driven and accelerated by a catalyst [123]. In other words, photocatalysis involves a solid material (photocatalyst) that absorbs light (photons) and induces a chemical reaction [124]. Photocatalysis is one of the Advanced Oxidation Processes (AOPs) that involves in-situ production of extremely potent chemical oxidants with the aid of Fenton’s reagent, hydrogen peroxide (H_2_O_2_), UV light, ozone (O_3_) or a catalyst. The produced hydroxyl radicals (∙OH) are strong enough to oxidize headstrong organic compounds [125,126]. AOPs are well-known methods for removal of CECs from wastewater effluent. Photocatalysis has extensively been studied by the scientific community for wastewater treatment, because of its ability to break down an ample range of organic materials, estrogens, dyes, organic acids, pesticides crude oil, microbes (counting viruses and chlorine resistant organisms), some inorganic molecules such as nitrous oxides and when combined with filtration or precipitation, it can also be used to remove metals (i.e., mercury) present in wastewater [127,128]. The nanomaterials display a different response compared to bulk materials, owing to their superior surface, mechanical, chemical, electrical, magnetic and optical properties, and distinct quantum effects and hence nanomaterials as a photocatalyst have recently gained great interest of researchers [129,130,131] [Figure 4].

Photocatalysis is a surface phenomenon and the mechanism associated involves five basic steps [132,133,134]:

- Diffusion of reactants/pollutants to the surface of photocatalyst
- Adsorption of reactants/pollutants on the surface of photocatalyst
- Reaction of adsorbed reactants/pollutants
- Desorption of products from the surface
- Removal/diffusion of products from interface

Some typically used nanostructured semiconductor photocatalysts are TiO_2_, Fe_2_O_3_, ZnO, zirconium dioxide (ZrO_2_), zinc sulfide (ZnS), tungsten trioxide (WO_3_), and cadmium sulfide (CdS) [135]. Bai et al. synthesized TiO_2_ loaded ordered mesoporous silica (SBA-15) molecular sieve deposited with zirconium nanophotocatalyst and evaluated its photocatalytic efficiency to degrade reactive red X–3B. The synthesized system was easily affected by pH variation and under optimal conditions it displayed 96% of degradation rate for reactive brilliant red X–3B dye [136]. Mahmoudian and coworkers fabricated silver (Ag) doped-TiO_2_ nanophotocatalyst for efficient degradation of recombinant DNA (rDNA) present in wastewater coming from a Hepatitis B surface antigen production plant. Upon evaluation, it was found that Ag-doped TiO_2_nanophotocatalysts have a great capability to degrade rDNA. Furthermore, calcination temperature and concentration of silver (Ag) had a significant effect on rDNA degradation. The rDNA degradation efficiency of synthesized nanophotocatalysts was found to be 80.7% [137].

As the use of antibiotics have increased, their presence in wastewater has also significantly increased. Ciprofloxacin (CIP) is one of the most widely used antibiotics globally and hence its occurrence in wastewater is increasing day by day. In order to remove CIP from wastewater, Malakootian et al. synthesized a heterogeneous magnetic nanophotocatalyst using carboxy methyl cellulose to remove CIP. The fabricated catalyst was characterized by its photocatalytic potential to remove CIP, its chemical stability and its reusability. The investigators found superior chemical stability, reusability and excellent CIP removal potential for designed magnetic nanophotocatalysts [138]. Similarly, Karimi and coworkers envisaged the synthesis of nanophotocatalysts using Fe_3_O_4_ NPs along with zinc sulfide quantum dots (ZnS-QDs), and N, S- doped graphene quantum dots (N, S-G@QDs) and compared its photodegradation ability with an organic dye pollutant, Victoria blue R (VBR), in aqueous media. The maximum photodegradation ability was found to be 96.68% for Fe_3_O_4_-N, S-G@QDs at pH 9, whereas Fe_3_O_4_-ZnS@QDs showed 93.44% of degradation at pH 8, after 120 min irradiation [139].

Occurrence of organic dyes such as MO, RhB and acid orange (AO7) have significantly increased in wastewater and their removal is a matter of concern. Yosefi et al. synthesized a p-n junction (formed by p type and n type semiconductor) flower-like nanophotocatalyst for removal of AO7 from wastewater. Bismuth oxyhalides (BiOX), namely, bismuth oxyiodide (BiOI), were used as p type and Zinc ferrite (ZnFe_2_O_4_) as n-type semiconductors. Fabricated P@BiOI/N@ZnFe_2_O_4_ showed 96% of degradation efficiency for AO7 in 3 h as compared to BiOI and ZnFe_2_O_4,_ alone [140]. Similarly, Margan et al. prepared ultrasound-assisted cadmium oxide-zinc oxide nanophotocatalyst (CdO-ZnO) for elimination of AO7. The photocatalytic degradation ability of this synthesized catalyst was found to be 69% within 140 min [141].

### 4.4. Disinfection and Pathological Control

According to the World Health Organization (WHO), 80% of diseases in developing countries are caused by consumption of water contaminated by pathogens including bacteria, viruses, prions, fungi, protozoa and rickettsia, that poses a serious threat to human health and take millions of lives each year around the world [142,143,144,145]. Disinfection is the process of reduction of the microbial count onto the surface or bulk of the material to an acceptable extent by physical or chemical means [146]. Traditional disinfection techniques adopted for wastewater treatment include free chlorine, hypochlorite, chlorine dioxide, chloramines, ozone, reverse osmosis and peracetic acid [147,148,149]. However, use of these techniques are limited because of their huge energy consumption, the need for expensive equipment and numerous DBPs, which raises other concerns [150,151]. Hence, there is an urgent need to develop efficient, sustainable, low-cost, inexpensive, less tedious and time saving disinfection methods [152].

**Figure 5:**
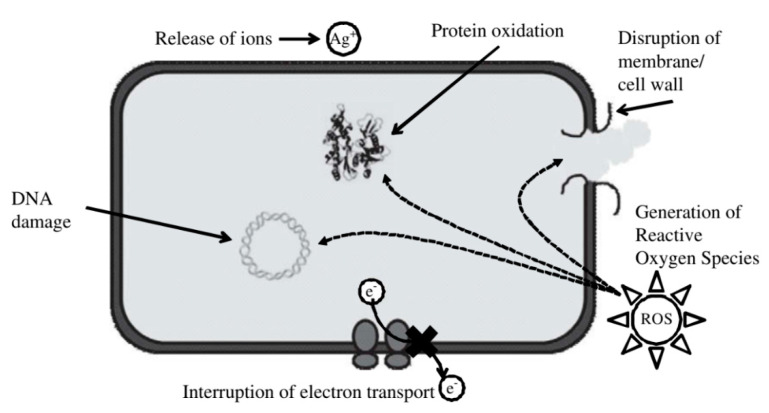
Schematic presentation of the different mechanisms of antimicrobial action of nanomaterials (Reproduced with permission from [161]).

Nanomaterials are endowed with elite functionality for inactivation of pathogens in water, such as large surface areas and specific reactivity, which cannot be achieved via conventional methods [153]. The inactivation mechanism of these nanomaterials include surface-based electrostatic interaction and photochemical reactions, which induces the generation of reactive oxygen species (ROS), the disruption of cell walls and targeted delivery of disinfecting agents [154,155,156,157] (Figure 5). Owing to great surface properties and the reactivity of these nanomaterials, inhibition of pathogens in water could be done easily [158]. Nanomaterials based on Ag, CuO, ZnO, TiO_2_, polymeric NPs and CNTs have been investigated for the disinfection of wastewater [159,160,161,162,163,164].

Ag has been known for its antimicrobial activity for more than a thousand years, and is still one of the most widely used NPs in microbial (bacteria, viruses, and fungi etc.) disinfection agents [165,166]. Consequently, reusable core-shell Ag@ZnO NPs have been developed in order to disinfect pathogenic bacteria counting *E. coli* and *Staphylococcus aureus*. The results evidenced complete elimination of both bacterial strains within 60 and 90 min of solar photocatalysis at 35 °C, respectively. Ag@ZnO core shell NPs in aqueous phase and higher efficiency was observed at 55⁰C temperature. The best possible mechanism of action of Ag@ZnO was proposed to be the generation of ROS during the catalysis, which leads to the damage of the bacterial cell walls [167]. ZnO NPs are one of the most biocompatible and environmentally friendly NPs [168]. To inactivate *E. Coli*, ZnO NPs were synthesized at varying pHs using surfactant-free reflux production techniques, which displayed potential antibacterial properties [169]. ZnO nanocrystal-doped macro mesoporous three-dimensional nanostructure silicon (Si)-wafers exhibited potential antibacterial activity for the model organism *E. coli.* The antibacterial activity of ZnO potentially increased in conjunction with Si-wafers [170].

Among the metal NPs, copper (Cu) NPs are some of the most effective and potent antimicrobial agents. One green synthesis approach for fabrication of antibacterial copper oxide (CuO) NPs (CuO NPs) was reported by Gul et al., where they fabricated NP CuONPs in the mixed matrices of PES and cellulose acetate by using casting techniques. Cu@pes-CA-CuO-1 (III) and Cu@PES-CACuO- 2 (IV) showed more than 75% inhibition, demonstrating excellent use of Cu as an antibacterial NP [171]. Another approach for synthesis of CuNPs from biowaste eggshell membrane was stated by He et al., using biotemplated methods and its antibacterial property was tested on model microorganisms such as *E. coli* and *S. aureus*. The antibacterial effect was indicated by the zone of inhibition for *E. coli* and *S. aureus*, which was found to be 20.3 and 27.5 mm, respectively. The synthesized CuO NPs showed a high recyclability [172].

### 4.5. Sensing and Monitoring

The environment has been contaminated by the presence of several living/non-living stuffs (e.g., disease causing microorganism, municipal/industrial waste, sewage discharge, animal defecation and heavy metals) [173]. Monitoring of water quality, on large as well as small scales, is a challenging task because of the extremely low concentrations of contaminants, complexity and variability of the wastewater matrices [174]. In order to address these issues, fast and efficient techniques need to be developed. In recent years, the scientific community have been more inclined towards the development of nanomaterial-based sensors to monitor water quality. Owing to their excellent properties, such as proficient recognition of trace contaminants, and fast analysis [8,175,176], nanosensors can be defined as device/material sensitive towards changes in surrounding stimuli, such as heat, chemical and mechanical stress, changes in volume, concentration, gravitational and magnetic, as well as electrical forces, and are used to convey physical, chemical or biological information about the behavior and characteristics of NPs from the nanoscale level to the macroscopic level [177,178]. Nanosensors consist of three main parts, namely a recognizing component (nanometals, nanotubes, nanowires, NPs, etc.) connected to a transducer (voltammetric, amperometric, conductometric, spectrophotometric, etc.) and a display for real time monitoring [179,180].

Biological contaminants such as antibiotic-resistant pathogens and their antibiotic-resistance genes (ARGs) are rising in wastewater continuously and this is daunting for public health. Methicillin-resistant *S. aureus* (MRSA) is a tarnished antibiotic-resistant pathogen whose mecA, an ARG of MRSA, is found to be responsible for antibiotic resistance. Riquelme and coworkers synthesized gold nanosensors functionalized with oligonucleotide for environmental monitoring of mecA ARG. The ARG spike detection test of synthesized nanosensors was performed in wastewater treatment plant effluent and it showed a high selectivity for ARG with the limit of detection of 70 ppm [181].

These days, organophosphorus (OP) compounds are being used widely as pesticides. As a result, their concentration in agricultural runoff, wastewater from industries and rivers has increased a lot. Since they are neurotoxic, their presence in water, even in traces, is harmful. Therefore, to detect OP compounds (diazinon pesticide) in tap water, agricultural runoff and rivers, Talari et al. designed aptamer-based optical nanosensors, utilizing reduced GO quantum dots (rGQDs) and MWCNTs. The nanosensors showed selectivity for diazinon and detected it promptly with a high accuracy [182]. Triclosan (TCS) is an antifungal and antibacterial agent widely used in household cleaning and personal care products. Extensive use of TCS and its subsequent release into wastewater, sediments and other water sources causes chronic toxicity to aquatic organisms as well as posing a risk to human health. Atar et al. developed a chemical nanosensor based on molecular-imprinted surface plasmon resonance (SPR) to detect TCS in wastewater. The developed nanosensor was applied to wastewater samples and the result revealed the excellent performance of the sensor [183].

Caffeine is a pharmaceutical and personal care product (PPCP), released abundantly in the environment by means of pharmaceutical wastewater, colas, tea, coffee beans, drugs and energy drinks. Hu et al. developed a chemical nanosensor based on AgNPs doped in molecularly imprinted polymers (MIP) for caffeine detection in wastewater. The detection limit of AgNPs@MIP for caffeine was found to be 100 ng L^−1^, which is less than the reported caffeine content in many rivers [184].

Cr is commonly used in steel manufacturing, painting, leather tanning, welding and as a catalyst as well. The extensive use of Cr has resulted in a tremendous increase in Cr contamination, which has perceptible adverse effects on biological and ecological systems. For the most part, Cr is found in two states of oxidation: trivalent chromium Cr (III) and hexavalent chromium Cr (VI). Cr (III) is an essential trace element for human nutrition, whereas Cr (VI) has mutagenetic and carcinogenetic effects on living organisms. In order to detect Cr (VI) in environmental water samples, Zhang et al. synthesized a carbon dot-based nanosensor. The synthesized nanosensor was found to be sensitive to Cr (VI) with the detection limit of 2.3 nM at pH 6 [185].

## 5. Barriers and Risks Associated with Nanotechnology

Although nanotechnology demonstrates promising outcomes in wastewater treatment, there are significant barriers that stand between these promises and their delivery. The most common barriers include nanomaterial toxicity, cost effectiveness and social acceptability. The risks associated include transformation of nanomaterials, ecotoxicity associated with engineered nanomaterials and water pollution [186].

### 5.1. Nanomaterial Toxicity

There have been many previous cases of wastewater treatment methodologies which have resulted in unwanted after effects. Chlorination is one of the conventional wastewater treatment methods which had been anticipated to contribute in enhanced life expectancy across the world, but was later noticed to generate carcinogenic byproducts like N-nitrosodimethylamine and trihalomethanes [187]. This could also be related to the utilization of nanotechnology in wastewater treatment. The properties which are responsible for the usefulness of nanomaterials are the ones which also make them liable for the resulting toxicity. Toxicity depends on the molecular structure of components dictating the toxicity end point and size, which regulates cellular uptake. Due to its small size, NPs penetrate through epithelial and endothelial barriers into the lymph and blood to various organs and tissues, including the brain, heart, liver, kidneys, spleen, bone marrow and nervous system [188]. Size- and shape-dependent toxicity is reported for Ag NPs, CNTs and many other metal NPs. The size of NPs (from 1 to 100 nm) are akin to the size of protein globules (2–10 nm), DNA helix (2 nm) and thicknesses of cell membranes (10 nm), allowing easy entry to cells and cell organelles [189].

**Figure 6:**
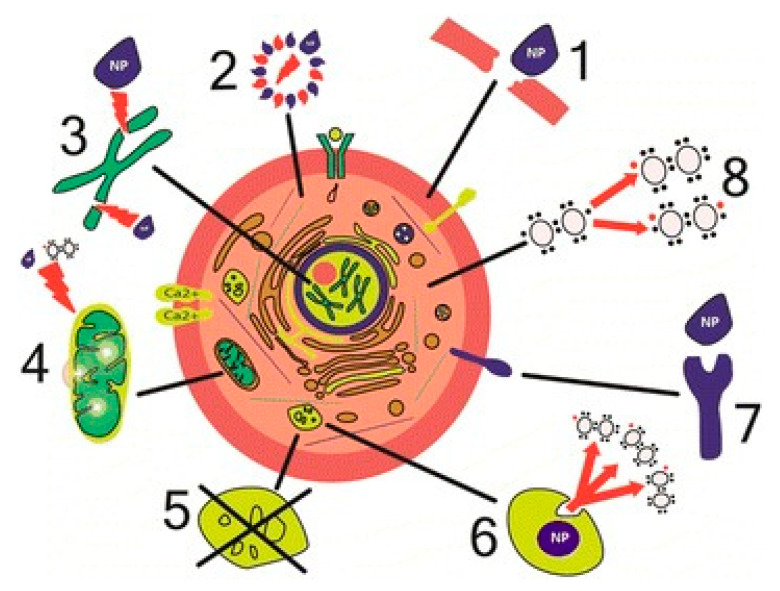
Mechanisms of cell damage by NPs. (1) Physical damage of membranes. (2) Structural changes in cytoskeleton components. (3) Disturbance of transcription and oxidative damage of DNA. (4) Damage of mitochondria. (5) Disturbance of lysosome functioning. (6) Generation of reactive oxygen species. (7) Disturbance of membrane protein functions. (8) Synthesis of inflammatory factors and mediators (Reproduced with permission from [189]).

Huo et al. reported that gold NPs of sizes less than 6 nm effectively enter the nucleus, while larger NPs of sizes 10–16 nm only penetrate through the cell membrane, and are thus found in the cytoplasm. TiO_2_ NPs are reported to make conformational changes in tubulin and inhibit its polymerization, thus disturbing intracellular transport, cell division and cell migration. NPs can be of different shapes, including spheres, ellipsoids, cylinders, sheets, cubes and rods. Spherical NPs are more prone to endocytosis than nanotubes and nanofibers. SWCNTs have been found to more effectively block calcium channels compared to spherical NPs. Hydroxyapatite NPs of different shapes, such as plate-like, rod-like, needle-like and spherical shapes, were evaluated for toxicity and demonstrated that plate-like and needle-like NPs resulted in the death of a larger proportion of nontumorigenic lung epithelial cells (BEAS-2B cells) as compared to spherical and rod-like NPs. Different mechanisms of cell damage by NPs are depicted diagrammatically in Figure 6.

An extensive toxicological database is available for bulk counterparts and shared constituents of nanomaterials benefit risk assessment. However, fullerenes and CNTs (allotropic nanomaterials) do not have bulk counterparts, which preclude such assessments and demonstrate the need for more vigilant toxicity studies. In a comprehensive prospective, a risk assessment should be considered at every stage in the life cycle of nanomaterials.

### 5.2. Cost Effectiveness

The performance and affordability of the nanotechnology for wastewater treatment eventually affects their acceptance. Developed countries use advanced technologies for wastewater treatment to remove wide spectrum of pollutants, while in developing countries it often covers the most basic needs (e.g., disinfectant). In both cases, there is a need to treat progressively complex contaminant mixtures to get a higher water quality at a lower cost, which pushes the boundaries of recent wastewater treatment models. Therefore, this cost barrier is significant but not impossible to overcome. A substantial fraction of the nanomaterial production cost is related to separation and purification. The cost of nanomaterials of research grade having high purity and uniform properties are having considerably constant prices from the last two decades and they are unlikely to drop significantly without increased demand and production scale-up. Moreover, the reasonability of using nanomaterials for wastewater treatment can be enhanced by producing nanomaterials of lower purity. For example, ultrapure C_60_ replaced with fullerene soot to make amino-fullerene photocatalysts, which exhibited a minimal loss of effectiveness with approximately 90% cost reduction [8,190]. Additionally, cost-effectiveness of nanomaterials can be enhanced by their long-term reusability. The example includes photocatalysts that retain its activity through the regeneration of nanoadsorbents, and magnetically separable multifunctional nanomaterials, which permits multiple reuse cycles [191].

### 5.3. Nanomaterial Transformation Risk in Water

Mode of interaction amid biotic–nanomaterials and abiotic factors, dispersibility/solubility governs the fate of nanomaterials. Generally, NPs settle slowly compared to larger particles, but because of their large surface area, they adsorb more sediment particles and soil, and due to their high insolubility in water (CNTs and fullerenes), they can easily be removed using water columns. The engineered nanomaterials are sometimes used for special purposes, i.e., pristine-engineered nanomaterials, which are prone to get transformed to various other forms, i.e., product-modified, product-weathered and environmentally transferred engineered nanomaterials. The light can bring out photochemical transformation and oxidation reduction can sometimes be favored. These transformations alter the interactions between nanomaterials and the environment, which eventually governs the adsorption and desorption of contaminants in water [192].

### 5.4. Ecotoxicity Associated with Nanomaterials

Ecotoxicity refers to the likeliness of chemical, physical or biological stressors to disturb the ecosystem. The nanomaterials have a potential risk of leaching into the treated water during production, use and discarding of NP-containing products. Moreover, there are higher production rates per year for emerging metal NPs like Ag and TiO_2_ [193]. These emerging nanomaterials are considered a great worry to the aquatic environment, as proved in one study where the inhibitory effects of Ag NPs and TiO_2_-NPs was investigated on the growth of duckweed (aquatic plant) [194].

NP concentrations in some natural surface waters are estimated to be on the nano/micro g/L scale. Theses concentrations increase with the enhanced production of NPs. Ag, titanium and zinc oxide NPs are used for their antibacterial properties to purify water in most developing countries, and replace chemical disinfectants [195]. Ceramic filters impregnated with Ag NPs are also used. Filtered water through nano Ag-coated filter papers showed detectable levels of Ag NPs, which were considerably below the limit set by the WHO’s guidelines [196]. Very few literature reports mentioned the studies investigating leached NPs from treated water and their toxicity on experimental animals. Some studies reported vital organ damage and DNA damage in rats, which were ingesting TiO_2_ in NP-contaminated water. Similarly, engineered nanomaterials are reported to initiate some health concerns, including pulmonary inflammation, genotoxicity, carcinogenicity and circulatory effects [197].

### 5.5. Water Pollution

Several laboratories work on toxicities caused due to nanomaterial-polluted water. Cimbaluk et al. evaluated the toxic effects of MWCNTs in two species of fish, *Astyanax altiparanae* and *Danio rerio,* where they find the possibility of CNTs-DNA crosslinking, the generation of oxidative stress and acute and subchronic neurotoxicity in species, respectively [198]. Similarly, Khan et al. studied the effects of Ag NP-treated water on fresh water fish (*Labeo rohita*), where they observed an elevation in oxidative stress and genotoxicity [199]. The toxicity is also reported to be due to the dispersant used for dispersing nanomaterials as they are poorly solubilized in water. For example, tetrahydrofuran is a very good dispersant used for CNTs and C_60_ fullerenes, which raises concerns about its toxicity [200].

The use of nanotechnology in wastewater treatment is subjected to systematic investigation of possible biological and ecotoxicity associated with their use. After ruling out any such possibility, the application of nanotechnology may result in promising outcomes in this arena, depending on the tactics being investigated by scientists to reduce the costs associated with nanomaterials. However, the initial results support the promising efficacy of nanotechnology in wastewater treatment and further optimization is supposed to increase their safety threshold.

## 6. Conclusions

Across the globe, the demand for clean and safe water is increasing with the rapid increase in population, expanding industrialization, urbanization and extensive agriculture practices. Various techniques are currently being used for the decontamination and purification of water. However, these methods often involve chemicals, and are energetically and operationally intensive, and hence require engineering expertise and infrastructure. Currently, there is a need for the development of novel wastewater treatment methodologies for the withdrawal of contaminants from wastewater. Nanotechnology could be one of the potential options in this aspect for nanomaterial-based wastewater treatment. Nanostructure materials are enriched with unique properties, such as high surface--to-volume ratios, high sensitivity and reactivity, high adsorption capacity and ease of functionalization. Owing to these properties, they have the potential to overcome the problems associated with traditional methods. However, another aspect of nanotechnology is the risk associated with it. Many issues have been reported by various researchers allied with a number of applications and properties of nanomaterials. Since they possess a very small size, they can be transmitted to human or other aquatic animal’s bodies and may cause toxicity. The further extent of toxicity greatly depends on surrounding conditions such as pH, concentration and contact time. Although scientists have explored nanotechnology a lot, a greater endeavor is obligatory in order to explore each and every corner of this new technology. The use of nanotechnology looks very promising for wastewater management and could have a great future in this regard, but a sincere and dedicated effort from the scientific community and government bodies is needed.

## Data Availability

Not applicable.
